# Check the box! How to deal with automation bias in AI-based personnel selection

**DOI:** 10.3389/fpsyg.2023.1118723

**Published:** 2023-04-05

**Authors:** Cordula Kupfer, Rita Prassl, Jürgen Fleiß, Christine Malin, Stefan Thalmann, Bettina Kubicek

**Affiliations:** ^1^Work and Organizational Psychology, Institute of Psychology, University of Graz, Graz, Austria; ^2^Business Analytics and Data Science-Center, University of Graz, Graz, Austria

**Keywords:** automation bias, elaboration likelihood model, experiment, dashboard, decision support systems, recruiting

## Abstract

Artificial Intelligence (AI) as decision support for personnel preselection, e.g., in the form of a dashboard, promises a more effective and fairer selection process. However, AI-based decision support systems might prompt decision makers to thoughtlessly accept the system’s recommendation. As this so-called automation bias contradicts ethical and legal requirements of human oversight for the use of AI-based recommendations in personnel preselection, the present study investigates strategies to reduce automation bias and increase decision quality. Based on the Elaboration Likelihood Model, we assume that instructing decision makers about the possibility of system errors and their responsibility for the decision, as well as providing an appropriate level of data aggregation should encourage decision makers to process information systematically instead of heuristically. We conducted a 3 (general information, information about system errors, information about responsibility) x 2 (low vs. high aggregated data) experiment to investigate which strategy can reduce automation bias and enhance decision quality. We found that less automation bias in terms of higher scores on verification intensity indicators correlated with higher objective decision quality, i.e., more suitable applicants selected. Decision makers who received information about system errors scored higher on verification intensity indicators and rated subjective decision quality higher, but decision makers who were informed about their responsibility, unexpectedly, did not. Regarding aggregation level of data, decision makers of the highly aggregated data group spent less time on the level of the dashboard where highly aggregated data were presented. Our results show that it is important to inform decision makers who interact with AI-based decision-support systems about potential system errors and provide them with less aggregated data to reduce automation bias and enhance decision quality.

## 1. Introduction

An increasing number of organizations use systems based on artificial intelligence (AI) to support decision-making in personnel selection (Black and van Esch, 2020). Many of those decision support systems focus on the preselection phase, e.g., resume screening (Lacroux and Martin-Lacroux, 2022). Such systems are ascribed multiple benefits for both organizations and applicants, such as a more efficient personnel selection process (Suen et al., 2019), as well as fairer and more accurate decisions (Oberst et al., 2021). AI collects, analyzes and visualizes data that is then presented in a dashboard to provide a solid decision base for first-party users, i.e., people who interact with the output of AI-based systems to make the selection decision, such as HR professionals (Langer and Landers, 2021). However, one major problem frequently reported with the use of AI-based decision support systems is the occurrence of automation bias, that is the thoughtless acceptance of decisions or recommendations made by the system (e.g., Mosier et al., 1996; Skitka et al., 1999; Parasuraman and Manzey, 2010; Goddard et al., 2012). Automation bias can lead to system errors being overlooked and thus result in poor decision quality (Mosier and Skitka, 1999; Bahner et al., 2008).

Despite the potential negative effects of automation bias on decision quality, little is known about the factors that might counteract thoughtless acceptance of AI-based recommendations in personnel preselection. Thus far, most research on AI-based decision support systems in personnel selection focuses on reliability and efficiency (e.g., Campion et al., 2016; Suen et al., 2020) or fairness perception and acceptance by applicants (e.g., Gonzalez et al., 2019; Langer et al., 2019; Acikgoz et al., 2020; Schick and Fischer, 2021; van Esch et al., 2021; for reviews see Langer and Landers, 2021; Hilliard et al., 2022). Only a few studies examine the decision makers of such systems in personnel selection (e.g., Langer et al., 2021; Li et al., 2021; Oberst et al., 2021; Lacroux and Martin-Lacroux, 2022). Those studies show that decision makers see the potential of a more efficient and fairer personnel selection process through AI-based systems (Li et al., 2021), while, at the same time, they seem to prefer recommendations from other HR professionals over those from an AI-based system (Oberst et al., 2021; Lacroux and Martin-Lacroux, 2022). Langer et al. (2021) demonstrated that proper timing of support from the system can influence decision makers` satisfaction with the selection decision as well as self-efficacy. However, factors that can minimize automation bias and, by doing so, increase the decision quality in the context of AI-based decision support systems for personnel preselection have to the best of our knowledge not yet been studied. In other contexts of AI-based decision support systems, strategies such as increasing decision maker responsibility, providing training and briefings, or having a group of humans as decision makers who monitor each other, have been investigated (Zerilli et al., 2019). An examination of these strategies in the personnel preselection context has yet to be conducted.

Providing empirical evidence on how to counteract automation bias and ensure a high decision quality in personnel preselection is important from ethical and legal perspectives. First, ethical standards call for human oversight of automation to address potential risks associated with AI use, e.g., system shortcomings (Hunkenschroer and Luetge, 2022). Second, the proposed European Union (EU) AI act (European Commission, 2021) requires human oversight in high-risk application areas of AI, such as personnel selection systems, which means a human investigation of each case and the possibility for decision makers to override AI recommendations. Moreover, Article 22 of the General Data Protection Regulation (GDPR) that applies in the EU states that “the data subject shall have the right not to be subject to a decision based solely on automated processing, including profiling, which produces legal effects concerning him or her […]” (The European Parliament and the Council of the European Union, 2016). For personnel preselection, this means that automatically processed information and AI-based decision-making must be reviewed by humans, a so-called human-in-the-loop, unless applicants voluntarily renounce their right. However, a passive approval of automated processing falls too short; human oversight rather needs to be an active assessment and verification (Malgieri and Comandé, 2017). Therefore, it is essential to investigate how decision makers can be encouraged to actively review AI-based recommendations in personnel preselection and meet these ethical and legal requirements, instead of blindly following decisions made by the AI.

Hence, we examine whether the way users are instructed about an AI-based system (i.e., receiving information about potential system errors and being made aware of the responsibility for the decision) as well as how the data is visualized (high versus low level of aggregation) has an impact on automation bias and decision quality. We do so by conducting an experimental study where participants made personnel preselection decisions with the help of a dashboard. We base our assumptions on the Elaboration Likelihood Model (ELM; Petty and Cacioppo, 1986), a dual-process theory that describes how human information processing can either follow the systematic central route or the heuristic peripheral route. We assume that organizational factors, i.e., the instruction about a system regarding errors and responsibility, and technological design factors, i.e., the level of data aggregation, can decrease the heuristic processing that is prone to automation bias and can increase decision quality.

Our study contributes to the literature on AI-based personnel preselection and automation bias in at least two ways. First, we provide recommendations on how AI-based decision support systems shall be introduced and designed to support decision makers to overcome automation bias and fulfil the legal requirement of human oversight. Only then such systems can be used to their full potential. Second, by integrating research on intelligent systems, automation bias, and ELM, we provide a solid theoretical basis to investigate which factors might counteract automation bias when interacting with AI-based decision support systems. This theoretical basis might also inspire future research on AI-based decision support systems in personnel preselection and other fields and business areas.

### 1.1. AI-based systems in personnel preselection

Technological advances in AI-technologies have led to their growing use in various business areas, including human resource management and personnel selection (Black and van Esch, 2020; Vrontis et al., 2022). In personnel selection, AI-based systems can support all phases of the selection process. AI-based systems can be used for the identification and attraction of potential candidates *via* databases and social media, for the screening and assessment of applicants *via* chatbots, video- or resume-analysis tools, and can administrate and communicate with applicants along the process (Hunkenschroer and Luetge, 2022). The most studied applications are chatbots (e.g., Eißer et al., 2020), resume screening tools (e.g., Acikgoz et al., 2020; Noble et al., 2021), and digital, highly automated video-interview tools that evaluate speech, facial expressions, and gestures (e.g., Langer et al., 2019; Acikgoz et al., 2020; Langer et al., 2021). The use of AI-based systems can make the laborious and time-consuming task of identifying and assessing potential candidates for decision makers easier and at the same time ensure a more consistent and fair decision-making process (Lacroux and Martin-Lacroux, 2022).

When it comes to the definition of AI, there is no consensus in the literature. AI is often used as an umbrella term for various approaches and techniques such as machine learning, deep learning or natural language processing (e.g., Pillai and Sivathanu, 2020). In the literature on AI in personnel selection, many authors follow machine learning approaches (e.g., Langer et al., 2021; Oberst et al., 2021; Kim and Heo, 2022) or have described AI as a technology that takes over tasks, particularly decision-making tasks, that previously required human intelligence (e.g., Acikgoz et al., 2020; Oberst et al., 2021; Pan et al., 2022). Following the initial idea by McCarthy et al. (1955), we define AI as the science and engineering of making intelligent machines. It seems that it is this vision that computers can do intelligent tasks that unites the research field (Moor, 2006).

Organizations use AI-based systems because they expect from them both efficient and impartial recommendations for selection decisions (Oberst et al., 2021; Lacroux and Martin-Lacroux, 2022). Hence, AI-based systems shall address a well-known challenge in personnel selection, namely the selection of applicants purely based on their qualifications and expected job performance, without (oftentimes unconscious and unintended) discrimination based on personal characteristics such as ethnicity (Quillian et al., 2017) or gender (Castaño et al., 2019). This places AI-based systems in the tradition of other decision support systems, such as paper-pencil tests, standardized interviews, or mechanical, algorithmic approaches, that have been shown to be clearly superior to so-called holistic methods, such as intuitive decisions by HR professionals (Highhouse, 2008; Kuncel et al., 2013; Nolan et al., 2016; Meijer et al., 2020). While a considerable increase in the practical use of AI-based systems for personnel preselection decisions is to be expected in the upcoming years, research is still in its infancy (Pan et al., 2022).

Systems to support human decision-making differ in their levels of automation, which refers to the balance of automation and human control in the decision-making process (Parasuraman et al., 2000; Cummings, 2017). Higher levels of automation provide fully automated decisions without a human decision maker involved. Lower levels of automation only provide recommendations as decision support and a human decision maker has control over which option is chosen (Cummings, 2017). As higher levels of automation in decision-making violate the legal requirements of Article 22 of the GDPR (The European Parliament and the Council of the European Union, 2016) and other ethical standards (Hunkenschroer and Luetge, 2022) that demand human oversight and thus a human who reviews the data and has control over the decision being made, we focus on AI-based decision support systems. One example of such a system that supports decision-making in personnel preselection *via* recommendations is a dashboard. A dashboard is defined as a data-driven system, which analyzes and visually presents data in a specific format to support decision-making (Yigitbasioglu and Velcu, 2012; Sarikaya et al., 2019). These visualizations of the data can have different designs and aim to extract information relevant to the decision. In the context of personnel preselection, data visualization by a dashboard means the analysis of the applicants’ data, including filtering irrelevant information, highlighting specific keywords, and assessing the applicants’ suitability for the job in form of a ranking list or a diagram (Raghavan et al., 2020; Langer et al., 2021). Relating to the field of visual analytics, it is essential how the analyzed data is presented or rather what data visualization format is chosen, to enable an effective information processing of the user (Cui, 2019).

### 1.2. Human information processing and automation bias

Due to ethical standards and regulations such as Article 22 of the GDPR, human oversight is demanded and, unless explicitly waived by applicants, legally required for AI-based personnel selection systems. Decision makers have to interact with the system to check recommendations and detect possible system errors. Previous research on AI-based decision support systems requiring human oversight highlighted the risk of automation bias (Cummings, 2017). Automation bias describes the tendency of people to thoughtlessly accept an automated decision or recommendation. Thus far, automation bias and its negative outcomes have primarily been investigated in aviation contexts (e.g., Mosier and Skitka, 1999; Davis et al., 2020) and medical contexts (e.g., Goddard et al., 2012; Lyell et al., 2018), but have also been found in the military domain and in process control (Bahner et al., 2008; Parasuraman and Manzey, 2010) as well as in quality control (Kloker et al., 2022). However, automation bias can occur in every work field that includes human-system-interaction (Goddard et al., 2012). In the case of AI-based personnel preselection systems, the occurrence of automation bias means that users do not review the data and actively make the decision, and hence the legal requirement of human control in a personnel selection decision is violated. It is thus crucial to investigate factors that might intensify verification behavior and thus prevent the occurrence of automation bias during the use of AI-based systems for personnel preselection.

To understand the origins of systematic distortions in human judgment, such as automation bias, it is important to take a closer look at human information processing. Several so-called ‘dual-process theories’ have described human information processing as a process with two distinct underlying systems (for an overview see Evans, 2011). These theories have great overlap in their theoretical foundations, however, we specifically base our assumptions on the ELM (Petty and Cacioppo, 1986), as it provides a comprehensive ground for our study and has been used to explain the acceptance of AI-based recommendations before (Michels et al., 2022). ELM describes how information processing occurs either *via* the peripheral route, which is characterized by fast, uncritical and heuristic information processing, or the central route, which describes thorough and systematic information processing. While the peripheral route is applied under time pressure or when limited or ambiguous information is available, the central route is engaged whenever decision makers have enough time and personal interest or motivation to critically process information (Petty and Cacioppo, 1986).

Automation bias aligns with the peripheral route of information processing according to the ELM. The users thereby use the automation’s recommendation as a heuristic replacement for thoughtful information seeking and processing (Mosier and Skitka, 1999). As this uncritical acceptance of system recommendations is to be avoided, it is imperative to promote information processing on the central route when using AI-based systems for personnel preselection.

### 1.3. Automation bias and decision quality

The use of AI-based systems, such as dashboards, in personnel preselection aims to enhance the efficiency and the quality of the decision-making process (Langer et al., 2021; Li et al., 2021). A high decision quality relies on the critical analysis of all applicants and the selection of the applicant, who best matches the job requirements (Kowalczyk and Gerlach, 2015; Langer et al., 2021). With regards to ELM, a systematical and critical elaboration of the applicants’ information is thereby crucial to ensure a high decision quality (Kowalczyk and Gerlach, 2015). Moreover, a dashboard serves as additional input for the decision maker, which helps mitigate the unconscious biases of the recruiter and increase the organization’s diversity. If the systems are used as assistance and recommendations are critically scrutinized, the additional input might disrupt fast and heuristic decision making and encourage the user to review hastily overlooked applicants more carefully (Raghavan et al., 2020; Li et al., 2021). Of course, this is only true if AI-based systems are not biased themselves. AI-based systems trained with insufficient or distorted data will fail to make correct predictions (Kim and Heo, 2022). However, the proposed European Union (EU) AI act (European Commission, 2021) aims to prevent these cases by setting quality criteria for training, validation and testing data sets for AI-based systems in high-risk areas, such as personnel selection. Optimally, the combination of human and AI-based information processing leads to a less biased and more thorough decision-making process (Langer et al., 2021).

One factor that affects user decision quality is the reliability and correctness of the system. If the decision recommended by the system is correct, users are more likely to efficiently make good decisions (Brauner et al., 2016). However, if the system’s recommendations are incorrect, the users’ decision quality is negatively affected. Users receiving incorrect advice show lower accuracy and longer decision times than people, who did not receive any support (Brauner et al., 2019). This impact on the decision quality can be explained by automation bias. Due to automation bias, users could either blindly follow the systems’ incorrect recommendation or check necessary information and still follow the incorrect advice of the system (Manzey et al., 2012). This means, that the users do not systematically elaborate the complete data but use the systems’ recommendation as a heuristic decision technique to avoid cognitive effort (Parasuraman and Manzey, 2010). Therefore, automation bias, including not seeking out confirmatory or contradictory information, can lead the user to follow a recommendation, even if it is not the best choice (Mosier and Skitka, 1999; Bahner et al., 2008), resulting in poor decision quality. On the other hand, decision makers who thoroughly process available information and thus exhibit a high verification intensity, should reach better decision quality.

*H1*: Verification intensity indicators are positively associated with decision quality when using an imperfect system.

### 1.4. Factors to mitigate automation bias and foster decision quality

**Figure 1:**
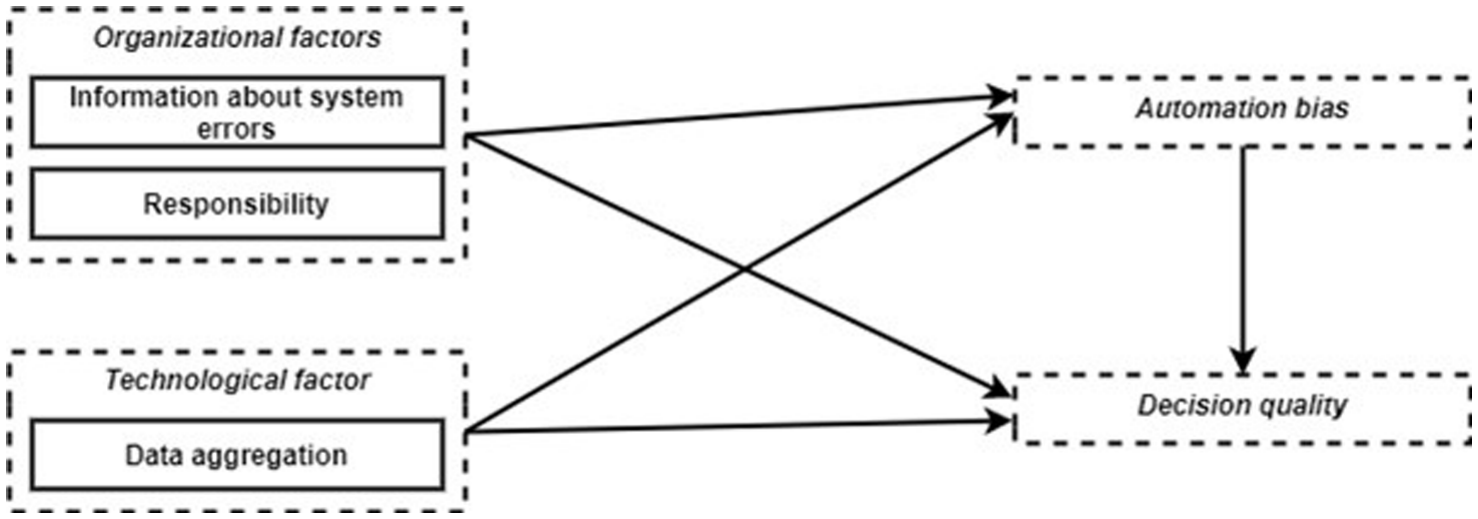
Proposed research model.

Several previous studies addressing the views of decision makers and AI-based personnel selection systems have identified technological, organizational, and environmental factors for successful deployment (e.g., Pillai and Sivathanu, 2020; Pan et al., 2022). However, those studies were cross-sectional surveys in companies on HR professionals’ perception of AI-based personnel preselection systems. They do not give us any information about the actual interaction with the systems during work processes and how good decision quality can be achieved. Additionally, automation bias has, to our knowledge, not yet been studied in the context of AI-based personnel preselection before. However, strategies to avoid automation bias have been tested in other application areas, especially in the aviation and medical context. It was found that responsibility for overall performance or decision accuracy can reduce automation bias in flight simulations (Skitka et al., 2000a). In another flight simulation study, joint decision-making in crews was compared with that of a single decision maker (Skitka et al., 2000b). However, team decision-making did not prove to be a suitable strategy to reduce automation bias; both crews and single decision-makers were equally subject to automation bias. In the same study, some participants were instructed about the phenomenon of automation bias and encouraged to verify the system. These participants performed better than participants in the control group and those who were prompted to verify the system (Skitka et al., 2000b). Other studies with process control tasks had the participants go through a training where they experienced that the supporting system was erroneous. This training led them to rely less on the system later in the test situation (Manzey et al., 2006; Bahner et al., 2008; Manzey et al., 2012). A review by Goddard et al. (2012) on automation bias and clinical decision support systems also emphasized responsibility, information and training as successful mitigation strategies. In addition, the design of the system, for example the dominant positioning of a recommendation on the screen, also had an impact on automation bias. In order to verify successful mitigation strategies also in the context of AI-based personnel selection, we conducted a work design study, focusing on organizational factors, i.e., information about system errors and responsibility, and technological design factors, i.e., the aggregation level of presented data (see Figure 1).

### 1.5. Information about system errors and automation bias

When introducing AI-based systems in personnel preselection contexts it can be crucial to inform users about possible system errors and make them reflect system recommendations more thoroughly. According to the ELM, the credibility of the information source has an impact on whether the presented information is either scrutinized or accepted uncritically (Petty and Cacioppo, 1986). In terms of using technology, the unawareness of the system’s capacities, i.e., its reliability, can lead to an overestimation of the systems’ credibility as users might heuristically decide to trust a system without systematically evaluating its capacities (Buçinca et al., 2021). The overestimation of the system’s capacities results in an inappropriately high level of trust and a heuristic reliance on the system, and thus enhances automation bias (Goddard et al., 2012; Buçinca et al., 2021). In line with that, prior research showed that increasing the users’ awareness of system errors and weaknesses can decrease automation bias: Users, who already experienced system errors during an initial training session, showed more verification behavior and thus less automation bias while later using the system (Manzey et al., 2006; Bahner et al., 2008). Consequently, making users aware of the systems’ capacity encourages them to process the systems’ recommendation more thoughtfully and control its recommendation more carefully. Therefore, users who are informed about potential system errors should show less automation bias in terms of higher verification intensity.

*H2*: Participants who are made aware of system errors score higher on verification intensity indicators than the control group.

### 1.6. Information about system errors and decision quality

More information about the AI-based system’s capacities, including its reliability, might stimulate a more critical investigation of the system’s recommendations, which positively influences decision quality (Bahner et al., 2008; Wickens et al., 2015; Sauer et al., 2016). As stated before, the unawareness of the system’s capacities might result in an overreliance on the system and thereby a heuristic acceptance of its recommendations (Parasuraman and Manzey, 2010; Buçinca et al., 2021). In the context of personnel preselection, decision makers might solely focus on best-ranked candidates while ignoring other lower ranked, but suitable candidates (Endsley, 2017; Langer et al., 2021). However, increasing the users’ awareness of the systems’ reliability and possible system errors might increase the users’ motivation to critically engage with all the available information (Bahner et al., 2008; Sauer et al., 2016; Endsley, 2017). This systematic information processing enhances decision quality as the decision maker verifies the systems’ recommendation and is less likely to follow a wrong recommendation (Parasuraman and Manzey, 2010; Kowalczyk and Gerlach, 2015; Buçinca et al., 2021).

*H3*: Participants who are made aware of system errors show a higher decision quality than the control group when using an imperfect system.

### 1.7. Responsibility and automation bias

Many guidelines, laws and regulations, such as the GDPR, demand human oversight and thus users must be made aware of their responsibility and accountability for the decision-making process and their obligation to monitor and control decisions from an AI-based system. Accountability and responsibility are two terms that are often used interchangeably but are in fact two distinct constructs. Accountability refers to a person’s obligation to explain and justify their decision and often arises from legislative or organizational sources. Responsibility, however, is more strongly related to the duty of completing a certain task and can be taken on by individuals themselves. In the context of personnel preselection, an HR professional is responsible for the task of selecting qualified personnel and he or she can be held accountable for the decision (Adensamer et al., 2021). We use the term responsibility in our study, as being held accountable for something also presumes being responsible for it in the first place.

One reason why automation bias might occur is the diffusion of responsibility mechanism. Diffusion of responsibility describes the psychological phenomenon of a decreased feeling of responsibility within a shared task as people unconsciously delegate their responsibility to their co-workers (Skitka et al., 2000a). Diffusion of responsibility also occurs in tasks humans share with automatic systems (Skitka et al., 2000a; Zerilli et al., 2019). Consequently, people who share a decision-making task with an AI-based decision support system, feel less responsible for the decision and reduce their cognitive effort. This leads to a more heuristic and peripheral information processing, which increases automation bias (Skitka et al., 2000a; Parasuraman and Manzey, 2010).

Conversely, people who feel responsible for the outcome of the decision tend to critically engage with and scrutinize the given information (Petty and Cacioppo, 1986). Skitka et al. (2000a) found that increasing the person’s responsibility for the decision can induce deeper information processing. People who were made responsible for the quality of the decision before the decision-making process engaged in more careful and deep information processing. This resulted in more verification behavior of the systems’ recommendation and thus decreased automation bias. Therefore, we propose that people who are made responsible for a decision show less automation bias in terms of higher verification intensity.

*H4*: Participants who are made aware of their responsibility for the decision score higher on verification intensity indicators than the control group.

### 1.8. Responsibility and decision quality

When sharing the selection task with an AI-based decision support system, decision makers might not attribute the decision outcome to their own effort (Nolan et al., 2016). This reduced feeling of responsibility may lead to a decrease in motivation, and cognitive effort and consequently impact the decision quality (Parasuraman and Manzey, 2010). According to the ELM, the feeling of responsibility increases the central processing of given information. Therefore, a stronger feeling of responsibility for the outcome of the decision should lead to a more critical engagement with the information, resulting in a more careful decision-making process and higher decision quality. In line with this argument, Skitka et al. (2000a) found that people who were specifically made responsible for the overall performance in a decision-making task made significantly better decisions than people who were not aware of their responsibility. Therefore, we propose that people who are made responsible for a decision show higher decision quality.

*H5*: Participants who are made aware of their responsibility for the decision show a higher decision quality than the control group when using an imperfect system.

### 1.9. Level of data aggregation and automation bias

Drawing from the field of visual analytics, the amount and format of the represented data of an AI-based system, such as a dashboard, can have a significant impact on how users process the information and how good the jointly reached decisions are (Endsley, 2017; Sosulski, 2018). Presenting too much data at one point can negatively impact the readability and understandability of the data visualization. The user might not be able to filter the relevant information and understand the key message of the visualization correctly (Sosulski, 2018). Conversely, presenting too little information, or information that is highly aggregated, can decrease transparency and limit critical elaboration of the data (Endsley, 2017; Sarikaya et al., 2019). Therefore, it is crucial to find the right level of data aggregation to enable an effective but reflected decision-making process.

AI-based data visualization refers to the dashboard’s capability to screen a big amount of data, summarize it and only present the most important information contained in the data (Parasuraman et al., 2000; Sarikaya et al., 2019). In the context of personnel preselection, this includes a summary of applicants’ qualifications and an assessment of their suitability for the position (Raghavan et al., 2020). Such a summary might be highly aggregated, presenting only an overall matching score of the candidates’ suitability or it might be less aggregated, presenting information on the candidates’ suitability in different areas such as qualification, abilities, and personality factors. According to the ELM, the presentation of strongly aggregated data might induce a more peripheral information processing, as presenting only specific parts of the data might lead users to pay less attention to the entire underlying data (Endsley, 2017). Moreover, the presentation of a specific recommendation, for example, a ranking list, might lead the users to solely focus on the AI-based recommendation, e.g., the best-ranked applicants (Langer et al., 2021). This means that the users reduce their information processing effort and use the systems’ recommendation as a heuristic to make a quick decision with relatively little cognitive effort. The reduced effort, however, increases automation bias (Parasuraman and Manzey, 2010; Onnasch et al., 2014). Thus, it can be argued that the display of more strongly aggregated data induces a heuristic information processing, which is expressed by accepting the recommended assessment without seeking and verifying background information, i.e., low verification intensity.

*H6*: Participants who see highly aggregated data visualizations score lower on verification intensity indicators than participants who see less aggregated data visualizations.

### 1.10. Level of data aggregation and decision quality

The format of data visualization affects how users interpret the underlying data and thereby influences their decision-making process (Endsley, 2017; Sosulski, 2018). Highly aggregated data, such as a single matching score, might on the one hand increase the users’ efficiency, as it provides a simple overview of the applicants’ suitability for the position (Langer et al., 2021). On the other hand, it decreases the users’ ability to validate the data. Therefore, a system error, i.e., an imperfect recommendation, might not be detected, resulting in the acceptance of a deflective decision (Parasuraman et al., 2000; Alberdi et al., 2009; Manzey et al., 2012).

Moreover, highlighting information and visualizing this information in a highly aggregated form can be problematic, as users tend to strongly focus on the highlighted information while ignoring contradictory information (Alberdi et al., 2009; Endsley, 2017). This means that users do not critically engage with the total information, but solely focus on information which the system deemed relevant (Parasuraman and Manzey, 2010; Endsley, 2017). Hence, presenting a highly aggregated summary of the candidates’ suitability for a job position might encourage a peripheral and heuristic elaboration of the presented data as not all data is taken into consideration, which decreases the soundness of the decision (Kowalczyk and Gerlach, 2015). Therefore, we propose that people who are presented with a highly aggregated data visualization, i.e., an overall matching score, show lower decision quality than people who are presented a less aggregated data visualization, i.e., a 5-point rating of three key indicators.

*H7*: Participants who see highly aggregated data visualizations show a lower decision quality than participants who see less aggregated data visualizations when using an imperfect system.

## 2. Methods

### 2.1. Research design

**Figure 2:**
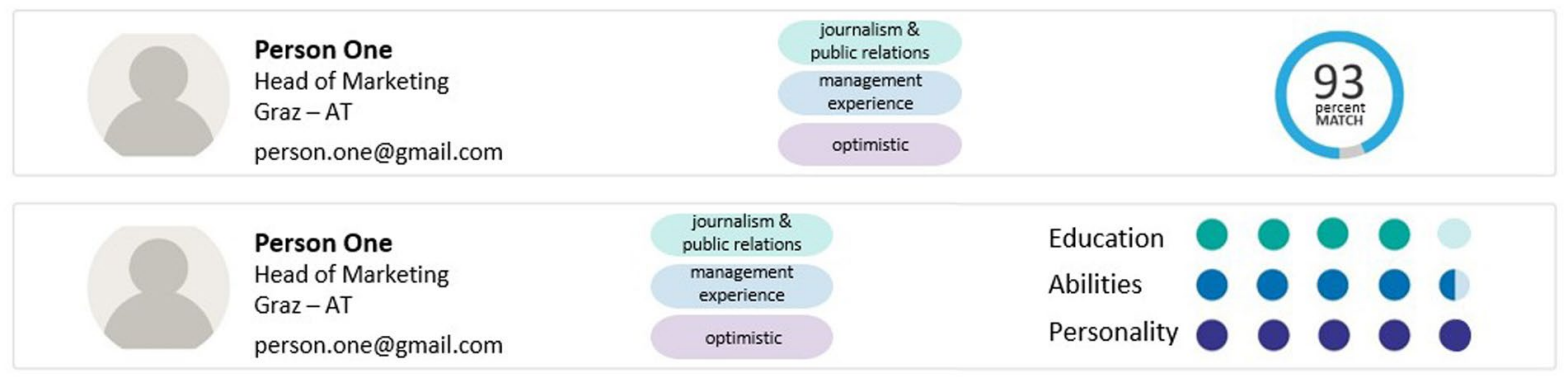
Dashboard level 1 – highly aggregated matching score (above) and low aggregated 5-point rating (below).

We conducted an experimental study using a 3 × 2 design, with the two between-subject factors system instruction (control group vs. error-awareness vs. responsibility) and data visualization (matching score vs. 5-point rating). The control group only received basic information about the dashboard and its functions. The error-awareness group additionally received more detailed information about the dashboard and a warning about possible system errors. The responsibility group received the basic information and information about their responsibility for the decision prescribed by the GDPR. They were told that they had to justify their decision at the end of the experiment. Table 1 provides the instruction texts of all groups. Concerning dashboard design (see Figure 2), the matching score group received an overall assessment of the candidates in form of a percentage score referring to the suitability of the candidates for the position. The other group received a 5-point rating of the candidates’ suitability concerning three key indicators, namely education, abilities and personality.

**Table 1:** Instruction presented to the participants in the different instruction conditions.

Condition	Instruction
Control group	This dashboard assesses applicants’ suitability for the position. The dashboard contains three different levels. The first level gives you an overview of all applicants, who applied for the position and their calculated suitability. The second level shows you more information about each applicant regarding their education, abilities, and personality. At the third level can access a protocol of a conversation between a chatbot and an applicant, in which the applicant answered questions about his or her personality. You can switch between the levels and the applicants any number of times.
Error-awareness group	[In addition to the information of the control group]. Applicants’ information has been processed and evaluated through artificial intelligence. The information has been extracted from the application using intelligent language processing. An algorithm compared this information with the job requirements and calculated applicants’ suitability. The calculation results are presented in the graphs. Prior studies have shown that when using similar systems, errors might occur. Therefore, it is essential to verify the dashboard’s assessment by checking all relevant information before decision-making.
Responsibility group	[In addition to the information of the control group] Please note that the dashboard is a decision support system and does not make the final decision. Article 22 of the General Data Protection Regulation (GDPR) states that subjects (here the applicants) shall have the right not to be subject to a decision based solely on automated processing. This means that you are obligated to verify the dashboard’s assessment. You are responsible for the selection decision. After the selection task, you will answer questions about the reasoning behind your selection decision.

Instruction texts are translated from German.

### 2.2. Participants

The sample size for the study was determined by an *a priori* power analysis using G*Power (Faul et al., 2009). Assuming a power of 1–β = 0.80, and an α-error of 0.05, we calculated a required sample size of 90 participants to be able to consistently detect medium-sized effects of *d* = 0.4. To account for potential dropouts, we recruited 100 participants *via* a student mail distribution list, flyers, and social media. Inclusion criteria for the study were the age of majority, the ability to understand German, and a general interest in personnel selection. Three participants were excluded from further analysis because they reported technical problems with the dashboard at the end of the study. After carefully checking the data, we removed an additional four participants as they had response times 1.5 SD below average in both experimental tasks. The final sample consisted of *N* = 93 participants (68% female and 2% diverse). The majority (90%) were full-time students, of which 82% studied psychology and 12% studied business administration. The remaining participants were full-time employees. The mean age was 23 years (SD = 3.89). Ten participants reported prior experience in human resource management. Psychology students received course credits for participating in the study.

### 2.3. Procedure

The study was conducted in a computer room at the university, where six participants could participate at the same time. Participants were randomly assigned to one of the six conditions. Participants were seated in front of a computer and were asked to read a printed written instruction. They were told to imagine themselves as HR professionals. Depending on the system instruction treatment, they either received basic information, information highlighting the potential error-proneness of the system or information highlighting the participants’ responsibility for the decision. After reading the instruction, participants had to complete two personnel preselection tasks for two different positions using either the low or highly aggregated ranking of the dashboard. Everyone completed the tasks in the same order. The first task was filling the position of head of the marketing department. The second task was filling the position of branch manager of a psychosocial facility. Participants were provided with a printed job description (see Supplementary material) with the requirements for each position. Participants had to select five out of ten applicants for each task and rank them according to how likely they would be to hire them. For each task, we intentionally included three errors. Two of the ten applicants were overrated by the dashboard, as they did not fulfil an essential requirement, while one applicant was underrated, because the dashboard did not recognize the applicant’s academic title (“Magister”). Participants could make their selection choice and end the task at any time but had a maximum of ten minutes to complete each task. After finishing the two experimental tasks, participants had to complete a series of questionnaires outlined in the measures subsection below. At the end of the experiment, the responsibility group received a debriefing, as they did not have to justify their decision as announced in the instruction.

### 2.4. The dashboard

**Figure 3:**
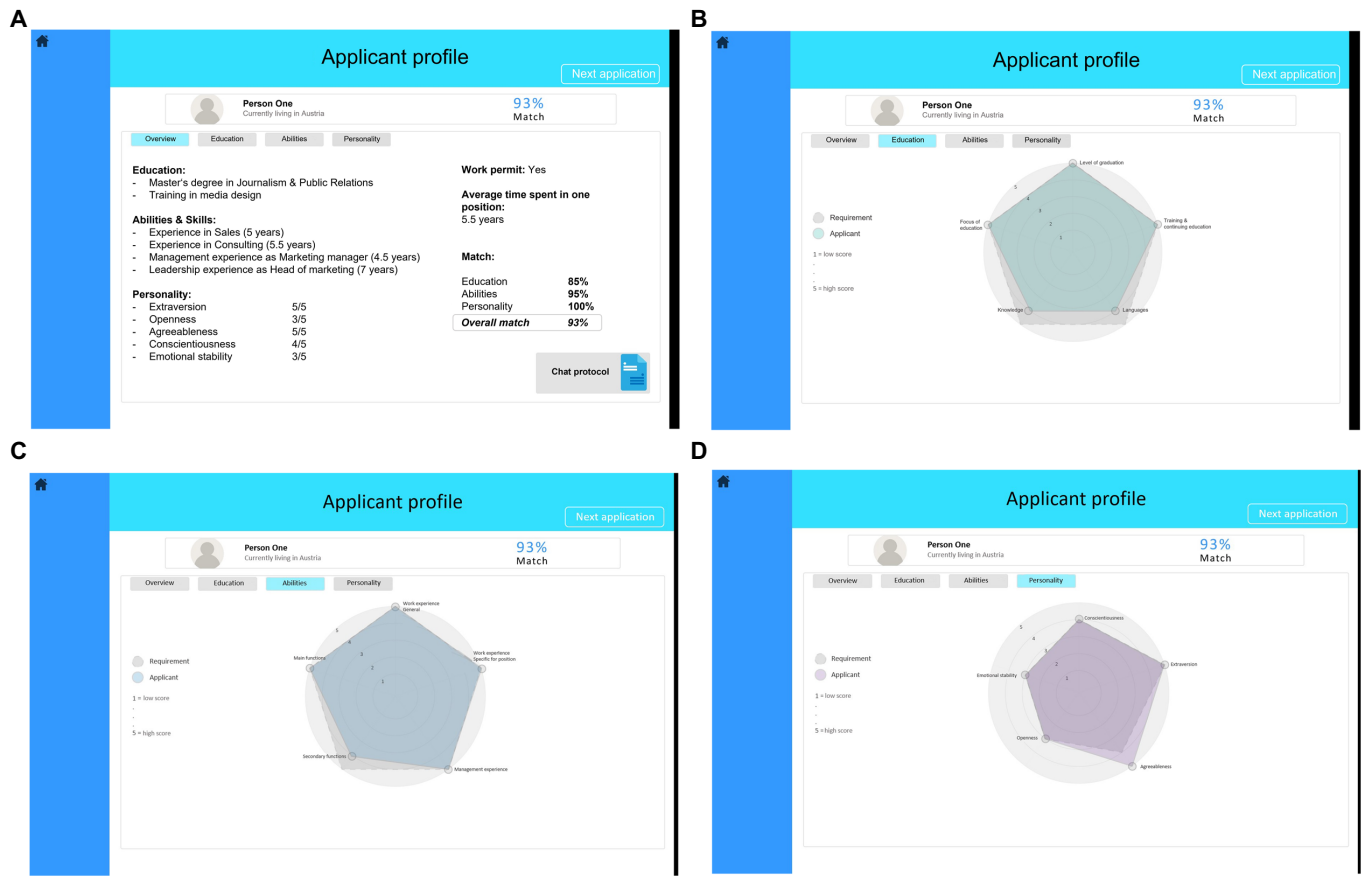
Dashboard level 2 – overview **(A)**, detail level education **(B)**, detail level abilities **(C)**, and detail level personality **(D)**.

**Figure 4:**
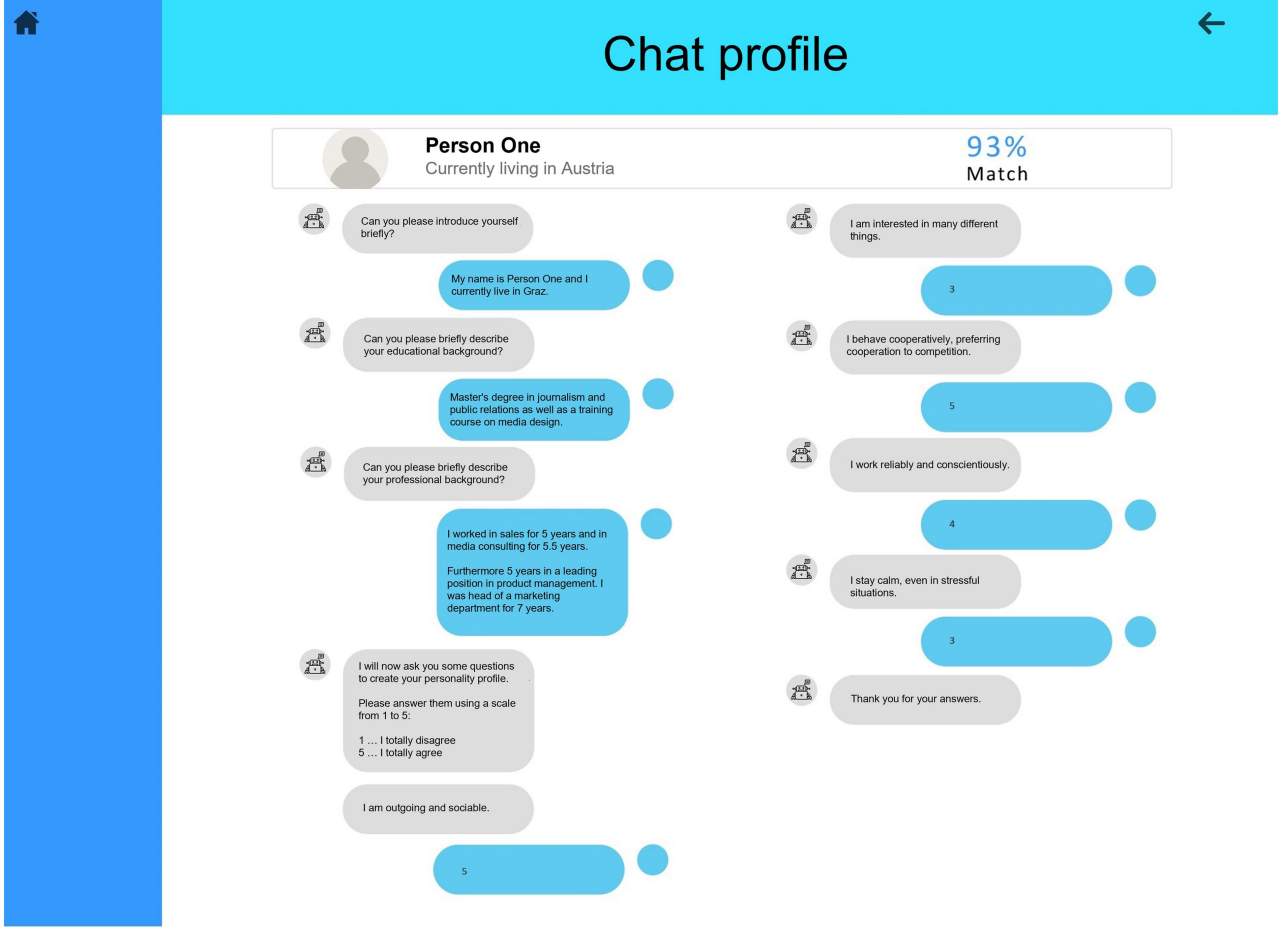
Dashboard level 3 – chatbot protocol.

The dashboard was designed with the software Preely (Testlab ApS, 2020). The main interface of the dashboard gave an overview of the ten applicants, an assessment of the applicants’ suitability for the position, and a few keywords from their CV (level 1, see Figure 2). By clicking on each applicant, the participants could access an overview of the applicants’ professional background (level 2, see Figure 3A). This overview contained more detailed information about the applicants’ education, prior work experience, and personality traits. From this interface, the participants could access an even more detailed interface for each key indicator (level 2 detail, see Figures 3B–D). The detailed interface contained a radar chart displaying how well the applicants match the job requirements regarding the key indicators. The dashboard was designed in such a way that the decision makers could quickly make a decision using the AI-based assessment at level 1, a realistic scenario in personnel selection. However, a decision based only on this assessment would mean that there would be no verification behavior by the decision makers. While level 1 provided an overview of all applicants, only level 2 provided enough information to thoroughly evaluate the applicants’ suitability for the position. Moreover, the integrated system errors could only be discovered at level 2. Therefore, level 2 must be accessed to verify the dashboard’s assessment. Proceeding from the level 2 interface, the participants could access a protocol of a conversation between a chatbot and the applicants, in which the applicants’ answers to questions from a personality inventory were displayed (level 3, see Figure 4). The participants were allowed to access every level and every applicant as often as they wanted. The dashboard was presented on 1,680 × 1,050 screens.

### 2.5. Measures

All measurements were administrated in German. Unless stated otherwise, all items were answered on a 5-point scale ranging from (1) *strongly disagree* to (5) *strongly agree*.

We included a manipulation check after the experimental tasks to verify if the experimental manipulation was effective. One item measured the effect of the warning presented to the error-awareness group. I controlled the dashboard’s assessment because I was aware that system errors might occur. Another item examined the feeling of responsibility. I controlled the dashboard’s assessment as I felt responsible for the selection decision due to the GDPR.

*Verification intensity indicators* were operationalized with three verification behavior variables, i.e., time spent at each level, the number of clicks, and the number of pages visited at each level during the decision-making process. Time, number of clicks, and visited pages were recorded with the software Preely (Testlab ApS, 2020).

*Decision quality* was measured in an objective and a subjective way. *Objective decision quality* was assessed by the number of correctly selected applicants. Five out of the ten applicants were designed to be better suited for each position than the other five applicants. Participants received one point for each correctly selected applicant, resulting in a possible score from 0 to 5. In addition, we assessed *subjective decision quality* by asking participants to rate their performance on the tasks using four self-developed items (see Table 2). A sample item is: “With the help of the dashboard, I selected the most suitable applicants.” The scale had an acceptable internal consistency (Cronbach α = 0.73).

**Table 2:** Scale for subjective decision quality.

Nr.	Item
1	With the help of the dashboard, I have invited the most suitable applicants for the position of head of the marketing department.
2	With the help of the dashboard, I have invited the most suitable applicants for the position of branch manager of a psychosocial facility.
3	I considered all the information before making a decision.
4	I accepted the dashboard assessments without checking them. (−)

Item Nr. 4 is inverted, high agreement means low subjective decision quality. All items are translated from German.

To control for confounding factors, we measured participants’ technical affinity and conscientiousness. *Technical affinity* was measured with the Affinity for Technology Interaction Scale (Franke et al., 2019). The questionnaire was answered on a 6-point scale ranging from (1) *strongly disagree* to (6) *strongly agree* (Cronbach α = 0.93). *Conscientiousness* was measured with the extra-short form of the Big-Five-Inventory-2 (Rammstedt et al., 2013). The scale had an acceptable internal consistency (Cronbach α = 0.76). In addition, we recorded participants’ gender (1 = female), age (in years), current occupation, highest education, and prior experience in human resource management (1 = yes).

## 3. Results

### 3.1. Manipulation check

To test whether the experimental manipulation of instruction was effective, we conducted two analyses of variance (ANOVA). The manipulation check showed that there was a significant difference between instruction groups concerning the awareness of system errors (*F*(2,57) = 7.47, *p* < 0.01, partial *η*^2^ = 0.13). Dunnett’s post-hoc tests revealed that participants of the error-awareness group (*M* = 4.03, SD = 1.10) were more aware of possible system errors than participants of the responsibility group (*M* = 3.00, SD = 1.39, *M*_diff_ = 1.03, 95% [0.26, 1.80], *p* = 0.01) or the control group (*M* = 3.00, SD = 1.44, *M*_diff_ = 1.03, 95% [0.21, 1.85], *p* = 0.010).

Moreover, it was shown that there was a significant difference between instruction groups concerning the feeling of responsibility (*F*(2,59) = 5.53, *p* = 0.02, partial *η*^2^ = 0.09). Dunnett’s post-hoc tests showed, that participants of the responsibility group (*M =* 3.50, SD *=* 1.24) had a stronger feeling of responsibility than participants of the error-awareness group (*M =* 2.70, SD *=* 1.26, *M*_diff_ = 0.83, 95% [0.04, 1.66], *p =* 0.036) and the control group (*M =* 2.64, SD *=* 1.28, *M*_diff_ *=* 0.86, 95% [0.05, 1.57], *p =* 0.03).

### 3.2. Verification intensity and decision quality

To examine hypothesis 1, stating that verification intensity indicators will be positively associated with decision quality when using an imperfect system, we conducted Pearson correlations. For this purpose, we correlated parameters of verification intensity indicative of automation bias, i.e., time spent, number of clicks, and visited pages with objective and self-rated decision quality.

For objective decision quality, there was a significant correlation between the verification intensity indicators and objective decision quality in both tasks for all level 2 interactions and for some level 3 interactions (see Table 3). In general, the longer the time spent, the greater the number of clicks and the greater the number of pages visited, the better the objective decision quality. Therefore, hypothesis 1 is partially supported in the case of objective decision quality.

**Table 3:** Pearson-correlations between verification intensity indicators and objective and subjective decision quality.

Variable	Objective decision quality task 1	Objective decision quality task 2	Subjective decision quality
*Time*			
Level 2	0.39**	0.35**	0.06
Level 2 detail	0.43**	0.28**	0.23*
Level 3	0.13	0.18	0.08
*Clicks*			
Level 2	0.39**	0.34**	0.18
Level 2 detail	0.40**	0.28**	0.21*
Level 3	0.12	0.23*	0.05
*Pages visited*			
Level 2	0.42**	0.36**	0.11
Level 2 detail	0.49**	0.34**	0.21*
Level 3	0.18	0.22*	0.10

*N* = 93; *p < 0.05, **p < 0.01 (two-tailed).

For subjective decision quality, there were only significant correlations between self-rated decision quality and the time spent (*r* = 0.23, *p* < 0.05), the number of clicks (*r* = 0.21, *p* < 0.05), and the number of pages visited (*r* = 0.21, *p* < 0.05) at the level 2 detail interfaces (see Table 3). Verification intensity indicators at other levels did not have significant associations with self-rated decision quality. Consequently, hypothesis 1 was partially supported for subjective decision quality.

### 3.3. Information about system errors, responsibility, and verification intensity

To test whether participants who were made aware of the occurrence of system errors (hypothesis 2) and responsible for the decision (hypothesis 4) show less automation bias in terms of higher verification intensity indicators than participants of the control group, we conducted a multivariate variance analysis (MANOVA) and subsequent ANOVA. We controlled for interactions between instruction and data aggregation conditions, but did not find significant interaction effects.^1^ For this purpose, we assessed the effect of the system instruction treatment on verification intensity indicative of automation bias, again including time spent on each level, the number of clicks, and the number of pages visited. Table 4 provides the means and standard deviations of the verification intensity indicators for each instruction group.

**Table 4:** Means and standard deviations of verification intensity indicators for each instruction group.

Task 1	Control group (*n* = 28)	Error-awareness group (*n* = 33)	Responsibility group (*n* = 32)
*Time (s)*			
Level 2	234.35 (185.29)	121.05 (144.65)	206.80 (163.89)
Level 2 detail	47.74 (76.67)	73.77 (61.56)	54.51 (66.82)
Level 3	27.47 (37.89)	35.95 (41.59)	24.47 (26.82)
*Clicks*			
Level 2	27.54 (20.57)	33.30 (23.35)	32.00 (26.41)
Level 2 detail	12.46 (18.93)	22.09 (17.72)	14.50 (15.19)
Level 3	1.71 (2.39)	3.09 (4.03)	1.84 (2.16)
*Pages visited*			
Level 2	7.43 (4.11)	8.12 (3.73)	7.44 (4.31)
Level 2 detail	8.32 (9.05)*	15.18 (11.06)*	10.47 (10.43)
Level 3	1.43 (1.85)	2.76 (2.93)	1.66 (1.86)
Task 2	Control group	Error-awareness group	Responsibility group
*Time (s)*			
Level 2	284.61 (190.24)	244.29 (135.80)	268.59 (172.96)
Level 2 detail	44.25 (62.69)	60.50 (54.45)	48.10 (67.10)
Level 3	15.22 (19.64) *	32.22 (31.97) *	17.77 (20.34)
*Clicks*			
Level 2	28.89 (20.69)	34.82 (23.86)	34.50 (29.84)
Level 2 detail	13.71 (20.01)	21.42 (18.28)	12.13 (13.07)
Level 3	1.32 (1.83)*	2.91 (2.92)*	1.91 (2.31)
*Pages visited*			
Level 2	8.25 (3.60)	8.58 (3.33)	8.22 (3.65)
Level 2 detail	8.43 (9.61)	14.76 (12.04)	9.91 (10.13)
Level 3	1.21 (1.62)*	2.33 (2.31)*	1.75 (2.00)

Time measures are expressed in seconds. *Means are significantly different at *p* < 0.05.

For the first task, time spent at each level (*F*(2,87) = 1.20, *p* = 0.18) and the number of clicks (*F*(2,87) = 2.69, *p* = 0.07) did not significantly differ between the instruction groups. However, a significant difference between the groups was found for the number of pages visited (*F*(2,60) = 3.59, *p* = 0.03, partial *η*^2^ = 0.07). The error-awareness group visited a larger number of pages at the level 2 detail interface (*M* = 15.18, SD = 11.06) than the control group (*M* = 8.32, SD = 9.05, *M*_diff_ = 6.86, 95% [0.67, 13.05], *p* = 0.03, *d* = 0.67). There was no significant difference between the responsibility group and the control group (*M*_diff_ = 2.15, 95% [−3.90, 8.20], *p* = 0.67).

For the second task, significant differences between the instruction groups were found for time spent at each level (*F*(2,59) = 3.35, *p* = 0.04, partial *η*^2^ = 0.09), the number of clicks (*F*(2,60) = 3.32, *p* = 0.04, *η*^2^ = 0.07), and the number of pages visited (*F*(6, 172) = 2.03, *p* = 0.06, *η*^2^ = 0.07). The error-awareness group spent significantly more time at level 3 (*M*_diff_ = 17.00, 95% [0.88, 33.12], *p* = 0.04, *d* = 0.63), had significantly more clicks at level 3 (*M*_diff_ = 1.59, 95% [0.11, 3.07], *p* = 0.04, *d* = 0.64) and visited significantly more pages at level 3 (*M*_diff_ = 1.12, 95% [0.07, 3.10], *p* = 0.04, *d* = 0.55) than the control group. These effects remained significant after adjusting for technical affinity and conscientiousness through analysis of covariance (*F*(2,88) = 3.48, *p* = 0.04, partial *η*^2^ = 0.07). However, there were no significant differences between the responsibility group and the control group with regard to time spent at each level (*M*_diff_ = 2.55, 95% [−9.88, 14.98], *p* = 0.88), clicks at each level (*M*diff = 0.59, 95% [−0.70, 1.87], *p* = 0.52), and number of pages visited (*M*_diff_ = 0.59, 95% [−0.70, 1.87], *p* = 0.52).

To sum up, decision makers of the error-awareness group tended to score higher on verification intensity indicators which means they expressed less automation bias. While there was a tendency for all indicators at all levels, only pages visited of the detail interface of level 2 during the first task and all indicators of level 3 in the second task differed significantly between the groups. Thus, hypothesis 2 is partly supported. However, the responsibility group did not significantly differ from the control group in their verification behavior. Thus, hypothesis 4 had to be rejected.

### 3.4. Information about system errors, responsibility, and decision quality

To test whether participants, who were made aware of the occurrence of system errors (hypothesis 3) and made responsible for the decision (hypothesis 5) show a higher decision quality than the control group when using an imperfect system, we conducted two ANOVAs, one for objective decision quality and another one for subjective decision quality. Again, we controlled for interactions between instruction and data aggregation conditions, which were all not significant.

For objective decision quality, i.e., the number of correctly selected applicants, no significant difference between the system instruction groups was found, neither in the first task (*F*(2,90) = 1.22, *p* = 0.30), nor in the second task (*F*(2,90) = 1.20, *p* = 0.32). In both tasks, the error-awareness group (*M*_task1_ = 4.39, SD*_task1_* = 0.86; *M*_task2_ = 4.39, SD_task2_ = 0.86) and the responsibility group (*M_task1_* = 4.16, SD*_task1_* = 0.72; *M*_task2_ = 4.19, SD_task2_ = 0.64) selected as many correct applicants as the control group (*M_task1_* = 4.10, SD*_task1_* = 0.74; *M*_task2_ = 4.46, SD_task2_ = 0.64). Thus, hypotheses 3 and 5 had to be rejected for objective decision quality.

However, for subjective decision quality, i.e., self-rated decision quality assessed at the end of the experiment, a significant difference between the instruction groups was found (*F*(2,90) = 4.08, *p* = 0.02, partial *η*^2^ = 0.08). Post-hoc testing revealed that the error-awareness group rated their decision quality significantly higher (*M* = 4.26 points, SD = 0.67) than the control group (*M* = 3.72 points, SD = 1.06, *M*_diff_ = 0.54, 95% [0.03, 1.01], *p* = 0.05, *d* = 0.62). This effect remained significant after controlling for conscientiousness through an ANCOVA (*F*(2,89) = 4.63, *p* = 0.01, partial *η*^2^ = 0.09). Consequently, hypothesis 3 was supported for subjective decision quality. Again, there was no significant difference between the responsibility group (*M* = 4.16 points, SD = 0.51) and the control group (*M* = 3.72 points, SD = 1.06, *M*_diff_ = 0.44, 95% [−0.10, 0.97], *p* = 0.13). Consequently, hypothesis 5 had to be rejected for subjective decision quality.

### 3.5. Data aggregation and verification intensity

To test hypothesis 6, postulating that participants who receive a more aggregated data visualization will show a stronger automation bias in terms of lower scores on verification intensity indicators than participants who are presented with a less aggregated data visualization, we conducted a MANOVA and subsequent *t*-tests for independent samples. For this, we examined differences in the verification intensity indicators, including time spent at each level, the number of clicks, and the number of pages visited between the data visualization groups.

For time spent at level 1, significant differences between the data visualization groups were found in the first task (*t*(78) = 2.74, *p* < 0.01, *d* = 0.57) and second task (*t*(69) = 2.10, *p* = 0.04, *d* = 0.44). The group, with a highly aggregated data visualization, spent significantly less time inspecting the level 1 interface (*M*_task1_ = 106.28 s, SD_task1_ = 99.97; *M*_task2_ = 62.96 s, SD_task2_ = 63.58) than the group with a 5-point rating of the key indicators (*M*_task1_ = 180.93 s, SD_task1_ = 157.43; *M*_task2_ = 105.78 s, SD_task2_ = 123.27). However, no significant differences in time spent at other levels, the number of clicks and the number of visited pages were found between the data visualization groups (*F*(3,85) = 0.23, *p* = 0.87), indicating no difference in verification intensity and thus the tendency of automation bias. Thus, hypothesis 6 was not supported.

### 3.6. Data aggregation and decision quality

To examine hypothesis 7 stating that participants who were presented with a more aggregated data visualization will have a lower decision quality than participants who were presented with a less aggregated data visualization when using an imperfect system, we conducted three *t*-tests for independent samples.

For objective decision quality, i.e., the number of correctly selected applicants, no significant difference between the data visualization groups was found in the first task (*t*(91) = −0.63, *p* = 0.53). The group with the overall matching score selected as many correct applicants (*M* = 4.17, SD = 0.74) as the group with a 5-point rating of the key indicators (*M* = 4.28, SD = 0.83). Similarly, no significant difference in the number of correctly selected applicants between the data visualization groups was found in the second task (*t*(91) = 1.09, *p* = 0.28). Again, the group with the overall matching score (*M* = 4.26, SD = 0.77) selected as many correct applicants as the group with a 5-point rating of the key indicators (*M* = 4.43, SD = 0.68). Thus, hypothesis 7 had to be rejected with regard to objective decision quality.

For subjective decision quality, i.e., self-rated decision quality assessed at the end of the experiment, no significant difference between the data visualization groups was found (*t*(91) = −0.03, *p* = 0.98). Participants who were presented with an overall matching score (*M* = 4.06, SD = 0.77) rated their decision quality equally well as participants assigned to the group with the 5-point rating of the key indicators (*M* = 4.05, SD = 0.82). Thus, hypothesis 7 also had to be rejected with regard to subjective decision quality.

## 4. Discussion

Given the importance of human oversight in AI-supported decision-making in high-risk use cases, this study focused on counteracting automation bias in the context of AI-based personnel preselection. We investigated how different organizational and technological design factors of an AI-based dashboard for personnel preselection influenced decision makers’ behavior concerning different verification intensity indicators and decision quality. Our experimental study showed that decision makers who scored lower on verification intensity indicators (i.e., less time spent on pages, lower number of clicks and pages visited), and thus had higher automation bias, selected fewer correct applicants. Lower scores on verification intensity indicators were associated with lower subjective decision quality. Organizational factors partially influenced verification intensity and decision quality: Information about system errors led in part to higher scores on verification intensity indicators and higher subjective decision quality, but unexpectedly not to higher objective decision quality. Contrary to our expectations, responsibility for the decision did not lead to higher scores on verification intensity indicators or higher objective and subjective decision quality. Data aggregation, as a design factor, did influence verification intensity at level 1 of the dashboard. Decision makers who viewed the more aggregated dashboard design spent less time at level 1 than those who viewed more detailed information at level 1. However, no differences in other verification intensity indicators and in objective and subjective decision quality were found.

Our study contributes to the literature on AI-based decision support systems by demonstrating the risk of automation bias in the context of AI-based personnel preselection. Automation bias has been found to lead to adverse effects on decision outcomes in several other contexts before (e.g., Parasuraman and Manzey, 2010; Goddard et al., 2012). This underscores the importance of identifying strategies to avoid this bias also in AI-based personnel preselection. To the best of our knowledge, our study is the first to explore strategies, that have previously been investigated in other application areas, such as raising responsibility and raising awareness about system errors (Zerilli et al., 2019), in the context of personnel preselection. If decision makers do not verify a system recommendation sufficiently, they might exclude suitable candidates from the personnel selection process, which is not only a loss for the organization, but also seriously affects candidates’ professional lives. From a legal perspective, these candidates could claim that they are being screened out by automated profiling due to insufficient human oversight.

Moreover, less verification intensity is also partially connected with lower subjective decision quality. This means, decision makers who do not check detailed candidate information and follow system recommendations, thus following heuristic information processing, do not believe in their own good performance, i.e., decision quality. Langer et al. (2021) also found, that decision makers who received an automated ranking of candidates before they even could process candidate information themselves, were less satisfied with their decision and had a lower feeling of self-efficacy compared to those who first processed candidate information and received an automated ranking later on. Possibly, decision makers who do not engage in thorough information processing along the central route, but rather engage the peripheral, heuristic route and follow system recommendations, do not feel they have contributed to the decision which could be reflected in dissatisfaction with decision quality. Our study thus indicates possible detrimental effects on decision makers supported by AI-based systems, that have been previously described in literature on AI-based system use. Burton et al. (2020) attribute the misuse of AI-based systems partly to unaddressed psychological needs of decision makers, like agency, autonomy or control.

Furthermore, our study contributes to research on automation bias by using the ELM (Petty and Cacioppo, 1986) to provide a solid theoretical foundation for understanding automation bias avoidance strategies. We found evidence that information about system errors influences decision makers’ verification intensity in the expected direction, with decision makers knowing about possible system errors seeking out more detailed information about candidates. Knowing that the system is not 100% reliable encourages users to critically check recommendations and to use the central, systematic route of information processing instead of the peripheral, heuristic route (Petty and Cacioppo, 1986). Previous studies demonstrated that decision makers who encountered system errors subsequently showed more verification behavior (Manzey et al., 2006; Bahner et al., 2008). Accordingly, the same experimental group, rated their subjective decision quality higher, which reflects the increased effort they put into decision-making. However, this group did not select more suitable applicants than the control group.

Contrary to our expectations, we did not find an effect of the responsibility condition on verification intensity indicators, objective and subjective decision quality. One potential explanation comes from prior research showing that performance improves when decision makers are responsible for the decision-making process, but not when they are responsible for the decision-making outcome (Doney and Armstrong, 1995; Siegel-Jacobs and Yates, 1996). Additionally, Skitka et al. (2000a) found in their studies that it is difficult to manipulate responsibility in experiments, as participants expect to be evaluated in experimental settings, partly due to instructions that are designed to encourage participants to take the experimental task seriously. Such evaluation concerns might have raised feelings of responsibility in addition to those elicited in the experimental group (that was informed about legal requirements due to the GDPR) and that were thus not captured by our manipulation check.

Lastly, we contribute to the literature on visual analytics (Cui, 2019) by providing an evaluation of different dashboard visualizations concerning the effect of data aggregation on automation bias in terms of verification intensity and decision quality. Decision makers who received a highly aggregated matching score spent significantly less time on the first level of the dashboard than the group who received the low aggregated 5-point rating of three key indicators. This finding suggests that the highly aggregated score did not convey sufficient information to fulfil the tasks, because participants of this group quickly switched to the other levels that presented more detailed information. However, this result could also reflect the cognitive effort required by decision makers to process more information in the less aggregated group compared to the highly aggregated group. We found no differences in other verification intensity indicators (i.e., on other system levels). This is in line with previous research, where users in a simulated process control task reduced the verification of additional parameters over time, but further controlled for necessary parameters (Manzey et al., 2012). In our study, decision makers of both groups were able to change levels and actively access more detailed information, so both groups were able to achieve the same decision quality. Therefore, no differences in objective and subjective decision quality were found between the high and low aggregated design. However, according to Yigitbasioglu and Velcu (2012) a good fit between data visualization and users’ information needs, as well as a balance between complexity and utility of the information visualization, are required to enable effective information processing by dashboard users. Our findings help to understand the needs of decision makers regarding the level of data aggregation in AI-based decision support systems. They suggest that highly aggregated information does not provide added value to decision makers and thus should be avoided.

### 4.1. Practical implications

As described above, the highly aggregated design did not lead to peripheral, heuristic information processing and, thus, less verification intensity. Instead, we observed that decision makers ignored level 1 of the dashboard with the aggregation score and searched for further information on other levels. These results emphasize that highly aggregated data alone are not enough for decision-making and that AI-based systems should give decision makers the option of accessing detailed information. To present information as parsimoniously as possible, we suggest that highly aggregated data should be avoided because they oftentimes do not convey sufficient information to reach a decision and can lead to oversimplification and automation bias.

In addition to technological design factors of AI-based personnel preselection tools, companies can adopt organizational strategies to reduce automation bias and promote high verification intensity. Bankins (2021) in her ethical framework for AI in human resource management proposes that organizations must align actual AI use with its intended use by instructing employees on how to interact and rely on AI. Based on our results we suggest that organizations inform decision makers about the actual capabilities of AI-based systems and raise their awareness of system errors to encourage high verification intensity. Since automation bias tends to occur especially when the system is perceived as highly reliable (Zerilli et al., 2019) and has been working error-free for a long time, i.e., has low failure rates (Parasuraman and Manzey, 2010), companies should make decision makers aware of possible system failures not once, but on a regular base. However, only informing users of possible errors might not reduce automation bias sufficiently. Experiencing system failures can have a stronger impact on user behavior (Bahner et al., 2008). Thus, an alternative strategy is to deliberately program errors into AI-based decision support systems and point them out when they are overlooked. This way, the design of the system can support the attention of decision makers by varying reliability over time (Goddard et al., 2012; Zerilli et al., 2019).

### 4.2. Limitations and future research

As with other research, this study is not without limitations. First, participants were students and not actual HR professionals. HR professionals might utilize a system for decision support differently, as prior experience with personnel selection tasks is related to higher confidence in one’s own decisions, resulting in a lower reliance on the system and less automation bias (Goddard et al., 2012; Langer et al., 2021). However, systems to aid decision-making might especially be considered for novice HR professionals as these systems tend to improve the decision-making quality of less experienced decision makers (Goddard et al., 2012; Langer et al., 2021). Therefore, we think that students are a suitable sample to reflect the target group of inexperienced HR professionals.

Second, the task was an isolated experimental task and not integrated into the stressful work situation of HR professionals. Automation bias often occurs in a multitasking setting and under a high workload, as it serves as a decision-making heuristic, which saves time and cognitive effort (Parasuraman and Manzey, 2010; Cummings, 2017). We have tried to simulate these conditions by limiting the available time for processing the tasks. In practice, the impact of interventions, i.e., the instruction and the data visualization, might affect the mitigation of automation bias more strongly. Future studies should investigate these interventions in real work settings.

Third, the personnel preselection task appeared to be rather simple, as the mean for objective decision quality, i.e., correctly selected applicants, was more than four points out of five possible points in all experimental groups. Our personnel preselection task only contained ten applicants per task. In a real-life personnel preselection task, a higher number of applicants can be expected, which increases the effort for information processing and makes decision-making more difficult (Black and van Esch, 2020). Possible impacts on the decision quality due to an unreflected use of the system as well as the uncritical acceptance of its recommendations might only arise under a higher workload (Parasuraman and Manzey, 2010). More complex tasks need to be explored in future studies.

In addition, future research should also have a closer look at individual differences between decision makers. We could observe high standard deviations for verification intensity indicators within experimental groups (see Table 4). Underlying differences at the individual level, like personality traits, individual information seeking styles or visualization preferences, could have influenced the verification intensity in addition to our experimental conditions. We controlled all analyses for consciousness and technical affinity, but found no differences. ELM also points to individual differences, such as the need for cognition, that influence information processing (Petty and Cacioppo, 1986). Future studies should thus consider other individual variables that could explain decision makers´ interaction with the dashboard.

When it comes to our finding that decision makers of the highly aggregated data group spent less time on level 1 than the less aggregated data group, it is yet unclear how this can be explained. The result could mean that highly aggregated data conveys too little information to be a support to the decision makers. However, it could also simply reflect the cognitive effort required by individuals to process the information presented. It is conceivable that people from the highly aggregated data group moved more quickly to other levels because they had less presented information to process than the other group. This open question should be considered by future research.

## 5. Conclusion

Automation bias has been found to be a serious problem in contexts of AI-based decision support systems (Mosier and Skitka, 1999; Goddard et al., 2012; Lyell et al., 2018; Davis et al., 2020), and violates ethical recommendations (Hunkenschroer and Luetge, 2022) as well as legal requirements like Article 22 of the GDPR (The European Parliament and the Council of the European Union, 2016) or the EU AI act (European Commission, 2021) that call for human oversight. Studies that previously examined AI-based personnel preselection tools from the perspective of decision makers have not yet addressed automation bias. An empirical investigation of automation bias in AI-based personnel preselection, and moreover strategies to avoid automation bias, is thus overdue. Our study confirmed that automation bias in terms of verification intensity influences decision quality in AI-based personnel preselection. Furthermore, we provide a first exploration of possible strategies to avoid automation bias in personnel preselection and provide first evidence that both organizational and technological design factors need to be considered when mitigating automation bias. Our study contributes to the literature by extending existing ELM and automation bias research to the context of AI-based personnel preselection tools.

## Data availability statement

The raw data supporting the conclusions of this article will be made available by the authors, without undue reservation.

## Ethics statement

Ethical review and approval was not required for the study on human participants in accordance with the local legislation and institutional requirements. The patients/participants provided their written informed consent to participate in this study.

## Author contributions

CK, RP, JF, CM, ST, and BK contributed to the conception and design of the study. RP collected data and performed the statistical analysis. CK and RP wrote the first draft of the manuscript. BK wrote sections of the manuscript. All authors contributed to manuscript revision, read, and approved the submitted version.

## Funding

This research was partly funded by the Field of Excellence Smart Regulation of the University of Graz. The authors acknowledge the financial support for open-access publication by the University of Graz.

## Conflict of interest

The authors declare that the research was conducted in the absence of any commercial or financial relationships that could be construed as a potential conflict of interest.

## Publisher’s note

All claims expressed in this article are solely those of the authors and do not necessarily represent those of their affiliated organizations, or those of the publisher, the editors and the reviewers. Any product that may be evaluated in this article, or claim that may be made by its manufacturer, is not guaranteed or endorsed by the publisher.

## Supplementary material

The supplementary material for this article can be found online at: https://www.frontiersin.org/articles/10.3389/fpsyg.2023.1118723/full#supplementary-material

## Footnotes

^1^There was only one significant interaction between instruction and data aggregation in task 1 on level 2 (*F*(6,172) = 2.16, *p* = 0.049, *η*^2^ = 0.04). However, as main effects were not significant and the effect size is small, we would be cautious to interpret this result.
