# Supplementary material for: Check the box! How to deal with automation bias in AI-based personnel selection
Source: Front Psychol. 2023 Apr 5;14:1118723. doi: 10.3389/fpsyg.2023.1118723 (PMC10113449; doi:10.3389/fpsyg.2023.1118723)
Supplement: Supplementary file 1 [file Data_Sheet_1.pdf]

Supplementary Material

Check the Box! How to deal with automation bias in AI-based personnel selection

Cordula Kupfer*, Rita Prassl, Jürgen Fleiß, Christine Malin, Stefan Thalmann and Bettina Kubicek

*** Correspondence:** Cordula Kupfer: cordula.kupfer@uni-graz.at

1 Supplementary Tables

To increase the reproducibility of this study, the next pages provide the two job descriptions used, one for the position of head of marketing department, the other for the position of branch manager of a psychosocial institution. Both job descriptions were translated from German.

Supplementary Table 1. *Job description for the position of head of marketing department.*

Personnel selection task 1

The position of **head of marketing department** of a large trading company is to be filled.

The candidate should have the following **education**:
- Academic degree in marketing & communication, journalism or business studies
- Knowledge of marketing tools and social media

The candidate should have the following **work experience**:
- Several years of work experience in marketing (preferably in a trading company)

The candidate should have the following **leadership experience**:
- Relevant professional experience in a management function (preferably in marketing or sales)

The candidate should have the following **qualities**:
- Strong communication and presentation skills as well as an outgoing personality (high extraversion)
- Structured and focused work style (high conscientiousness)
- Empathic personality as well as a strong team player (high agreeableness)

Required personality profile:
Extraversion: 5/5
Openness: 3/5
Agreeableness: 4/5
Conscientiousness: 3/5
Emotional stability: 3/5

Since the position must be filled as soon as possible, the candidate should already have a **work permit in Austria.**

As frequent personnel changes stress the working atmosphere, it is planned to fill the position for a longer period of time, which is why candidates **without frequent career changes** are wanted.

Note. *The job description is translated from German.*

Supplementary Table 2. *Job description for the position of branch manager of a psychosocial institution.*

Personnel selection task 2

The position of **branch manager of a psychosocial facility** is to be filled.

The candidate should have the following **education**:

- Academic degree in social pedagogy, psychology or an equivalent education for social professions
- Preferably additional qualifications in counseling

The candidate should have the following **work experience**:

- Several years of work experience in assisting people with mental illnesses and/or disabilities

The candidate should have the following **leadership experience**:

- Several years of management experience in a psychosocial facility or a pedagogical facility for disabled people

The candidate should have the following **qualities**:

- High social competence and ability to deal with conflicts (high agreeableness)
- Strong teamwork and collaboration skills (high agreeableness)

Required personality profile:
Extraversion: 4/5
Openness: 2/5
Agreeableness: 5/5
Conscientiousness: 4/5
Emotional stability: 4/5

Since the position must be filled as soon as possible, the candidate should already have a **work permit in Austria.**

As frequent personnel changes stress our clients, it is planned to fill the position for a longer period of time, which is why candidates **without frequent career changes** are wanted.

Note. *The job description is translated from German.*
